# Tracking Transmission of Apicomplexan Symbionts in Diverse Caribbean Corals

**DOI:** 10.1371/journal.pone.0080618

**Published:** 2013-11-19

**Authors:** Nathan L. Kirk, Raphael Ritson-Williams, Mary Alice Coffroth, Margaret W. Miller, Nicole D. Fogarty, Scott R. Santos

**Affiliations:** 1 Auburn University, Department of Biological Sciences and Molette Biology Laboratory for Environmental and Climate Change Studies, Auburn, Alabama, United States of America; 2 Smithsonian Marine Station, Fort Pierce, Florida, United States of America; 3 State University of New York at Buffalo, Department of Geology, Buffalo, New York, United States of America; 4 National Oceanic and Atmospheric Administration, Southeast Fisheries Science Center, Miami, Florida, United States of America; 5 Oceanographic Center, Nova Southeastern University, Dania Beach, Florida, United States of America; 6 Cellular & Molecular Biosciences Peak Program, Auburn University, Auburn, Alabama, United States of America; Pennsylvania State University, United States of America

## Abstract

Symbionts in each generation are transmitted to new host individuals either vertically (parent to offspring), horizontally (from exogenous sources), or a combination of both. Scleractinian corals make an excellent study system for understanding patterns of symbiont transmission since they harbor diverse symbionts and possess distinct reproductive modes of either internal brooding or external broadcast spawning that generally correlate with vertical or horizontal transmission, respectively. Here, we focused on the under-recognized, but apparently widespread, coral-associated apicomplexans (Protista: Alveolata) to determine if symbiont transmission depends on host reproductive mode. Specifically, a PCR-based assay was utilized towards identifying whether planula larvae and reproductive adults from brooding and broadcast spawning scleractinian coral species in Florida and Belize harbored apicomplexan DNA. Nearly all (85.5%; *n* = 85/89) examined planulae of five brooding species (*Porites astreoides, Agaricia tenuifolia, Agaricia agaricites, Favia fragum, Mycetophyllia ferox*) and adults of *P. astreoides* were positive for apicomplexan DNA. In contrast, no (*n* = 0/10) apicomplexan DNA was detected from planulae of four broadcast spawning species (*Acropora cervicornis*, *Acropora palmata*, *Pseudodiploria strigosa*, and *Orbicella faveolata*) and rarely in gametes (8.9%; *n* = 5/56) of these species sampled from the same geographical range as the brooding species. In contrast, tissue samples from nearly all (92.0%; *n* = 81/88) adults of the broadcast spawning species *A. cervicornis*, *A. palmata* and *O. faveolata* harbored apicomplexan DNA, including colonies whose gametes and planulae tested negative for these symbionts. Taken together, these data suggest apicomplexans are transmitted vertically in these brooding scleractinian coral species while the broadcast spawning scleractinian species examined here acquire these symbionts horizontally. Notably, these transmission patterns are consistent with those of other scleractinian coral symbionts. While this study furthers knowledge regarding these symbionts, numerous questions remain to be addressed, particularly in regard to the specific interaction(s) between these apicomplexans and their hosts.

## Introduction

 Symbioses, defined here as the intimate association of two different organisms [1], have helped shape the evolution of eukaryotic life [2] and the ubiquity [3,4] and antiquity [5,6] of these relationships demonstrates their widespread success in general. Of importance to any symbiosis is continuity across generations. In this context, symbionts may be passed vertically from parents to offspring or acquired horizontally via a vector or from the local environment. For the symbiont, there is direct benefit from vertical transmission as a new host individual is guaranteed. However, the fate of the symbiont is often tied to the local extirpation or extinction of their host species in strictly vertical systems [7–9]. On the other hand, horizontal transmission includes the uncertainty of whether suitable partners will encounter each other in subsequent generations. Given that vertical or horizontal transmission have potential pitfalls for either (or both) of the partners, it is not surprising that the specific mode varies between hosts and their various symbionts and that symbionts can be acquired through multiple routes. For many symbioses, however, the transmission mode of particular symbionts remains to be elucidated.

 Serving as the foundation of the tropical reef ecosystem, scleractinian corals within the phylum Cnidaria provide services such as nutrition and shelter to a wide-range of other organisms [10,11]. Scleractinian corals are an ideal system to study modes of transmission as they harbor numerous, diverse symbionts. Specifically, corals form symbioses with members from all three domains of life: Eubacteria, Archaea, and Eukaryota [12], with their most well-known relationship involving dinoflagellates in the genus *Symbiodinium*, which translocate photosynthetically-fixed carbon to the host [13]. Along with *Symbiodinium*, other mutualists and parasites of scleractinian corals influence host health and physiology in both positive and negative ways [14–18].

 Given the importance of symbiont assemblages to scleractinian corals, considerable work has been conducted towards understanding their transmission dynamics. Generally, symbiont transmission in scleractinian corals is related to the reproductive mode of the host species [19,20], with different modes dependent upon whether syngamy occurs internally or externally of the maternal colony. For example, species possessing internal fertilization produce planula larvae (hereafter referred to as planulae) that develop within the maternal colony prior to release, termed “brooding”. These host species tend to provision symbionts like *Symbiodinium* vertically [20]. Conversely, species releasing gametes into the water column, in a process called “broadcast spawning”, have external fertilization and the resulting planulae most often obtain symbionts, such as *Symbiodinium*, horizontally as larvae or upon settlement and metamorphosis [20–22]. Both possible modes of transmission appear to broadly apply across a wide taxonomic range of symbionts, from Eubacteria [23–25] to eukaryotic stramenopiles (Protista: Chromista) [26].

 Here we focus on elucidating the transmission mode of another group of scleractinian coral symbionts, the eukaryotic apicomplexans. Evolutionarily, this clade of ~6,000 described species is sister to the dinoflagellates and almost exclusively comprised of parasites, including the causative agents of malaria and toxoplasmosis [27,28]. The first Apicomplexans documented from coral hosts were described as a single species, *Gemmocystis cylindrus*, based on morphology and life-cycle [29,30]. Subsequently, apicomplexans and apicomplexan-related lineages (ARL) have been detected in numerous scleractinian corals and gorgonians using various genetic approaches [31–36]. However, the impacts these symbionts have on their scleractinian coral hosts, such as fitness costs, remain unknown. Furthermore, their transmission mode among host individuals, which could be vertical, horizontal or both, remains unresolved. This study examined the gametes, planulae, and adults from multiple species of brooding and broadcast spawning scleractinian corals from reefs in both the Florida Keys and Belize towards elucidating transmission mode(s) of these under-recognized, but apparently widespread, coral-associated symbionts.

## Methods

### Ethics Statement

 Collection of all scleractinian coral gametes, planulae and adult tissues was permitted through appropriate regulatory bodies and in accordance to the permits and laws of the issuing body. Specifically, Florida samples were collected in accordance to the following permits from the Florida Keys National Marine Sanctuary: 2010 *Porites astreoides* adult and larvae colonies: (FKNMS–2010–039); 2011 *P. astreoides* larvae: (FKNMS–2010–023): broadcast spawning gametes and larvae (FKNMS–2009–081–A and FKNMS–2010–055). In Belize, all colonies and larvae were collected by permit from the Belize Fisheries and imported according to CITES permits (131, 385, 1817, 1818).

### Collection of Planulae and Adults from Brooding Species

**Figure 1:**
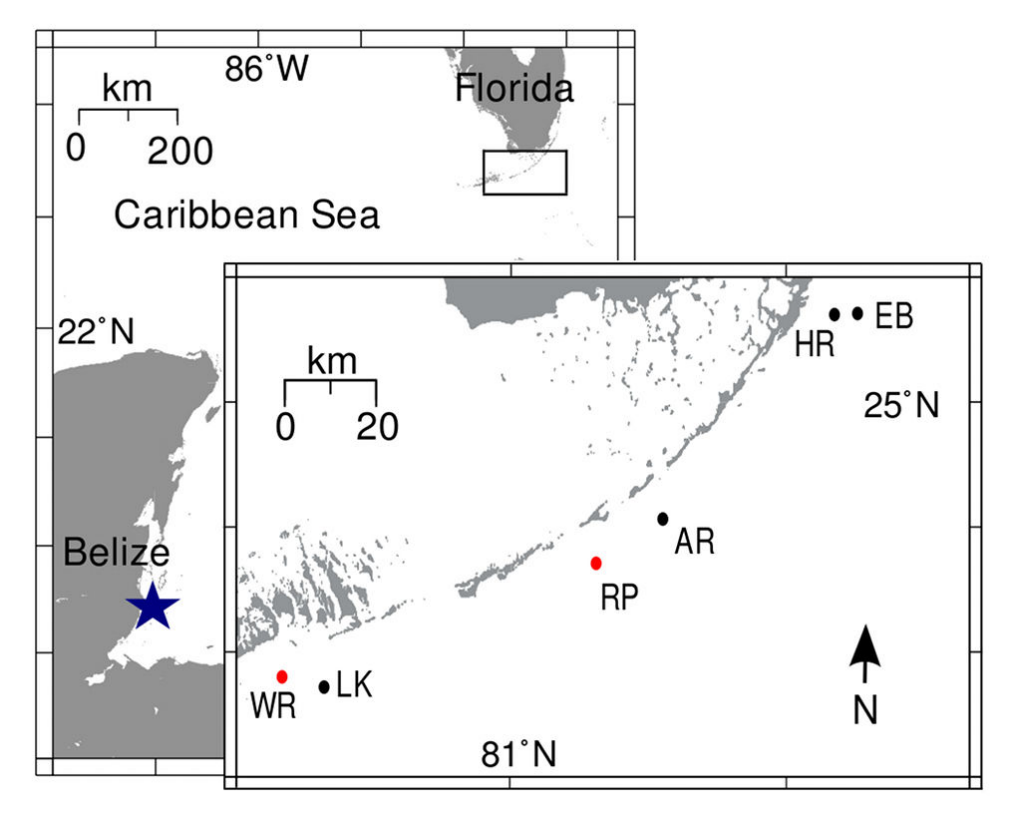
Map of coral reefs sampled in this study. Inset provides finer scale regional resolution for reefs in the Florida Keys. Brooding scleractinian coral species were collected at reefs indicated in red while broadcast spawning species were collected at reefs indicated in black. Reefs denoted in blue represent sites where species from both reproductive modes were collected. Elbow Reef=EB, Horseshoe Reef=HR, Alligator Reef=AR, Rubble Piles=RP, Looe Key=LK, Wonderland Reef=WR. See Tables 1 and 2 for more detail.

 In May 2010, 30 colonies of the brooding scleractinian coral *Porites astreoides* were collected from an artificial patch reef established in 1986 in the Middle Keys (Bureau of Marine Fisheries Management (1999); Rubble Piles [RP]: N 24.742778°, W 80.814722°, Figure 1). Larger (13.1 +/- 2.2 cm^2^ in diameter) colonies were chosen to maximize reproductive probability [37] and collected 3 days prior to the new moon when *P. astreoides* was predicted to release larvae [37,38]. As few colonies released planulae in 2010 (see Results), the experiment was repeated in 2011 in the lower Florida Keys (Wonderland Reef [WR]: N 24.56028°, W 81.50127°, Figure 1). There, fifty-one *P. astreoides* colonies were collected in April 2011, five days prior to the new moon (Figure 1). For both years, colonies were placed into collection buckets daily and prior to dusk [39,40] and released brooded planulae over subsequent nights. Planulae were collected the morning of first release and preserved in 95% ethanol for molecular analyses. To determine whether apicomplexans were present in all planulae or just in those released on the first day, collections were made from five maternal colonies over three consecutive days, which was the duration of the April 2011 reproduction event. Additionally, ~1.0 cm^2^ tissue samples were removed from the edge of all maternal colonies in 2010 and preserved as above to test whether these reproductive adults harbored apicomplexan symbionts. 

 To determine if apicomplexans were present in planulae of additional Caribbean brooding corals, 15–40 colonies from four other species as well as *P. astreoides* (Table 1) were collected from reefs surrounding Carrie Bow Cay (CBC: N 16.80250°, W 88.08194°, Figure 1) on the Belizean Barrier Reef and kept in individual collection buckets as described above. Specifically, *P. astreoides* and *Agaricia tenuifolia* colonies were collected on the day of the new moon (when both were predicted to spawn) from the reef flat directly adjacent and northeast of CBC and from a small patch reef ~200 m north of CBC, respectively. The following day, colonies of *Mycetophyllia ferox* and *Agaricia agaricites* were collected from the fore-reef ~200 m east of CBC. Colonies of *Favia fragum* were collected on 8 June and again on 10 August from the same patch reef as *A. tenuifolia*. This species was collected later in the lunar cycle when individuals have a higher likelihood of releasing brooded planulae [41]. All planulae were collected the morning following release and preserved as described above. 

**Table 1:** Information for the brooding scleractinian coral species sampled in this study from Florida and Belize.

**Species**	**Locat^1^**	**Depth**	**Collection Date^2^**	**Release Date^3^**	**No. A^4^**	**No. R^5^**
*P. astreoides*	Florida	4-6 m	12 May, 2010	12-16 May	30	14
*P. astreoides*	Florida	4-7 m	30 April, 2011	30 April- 2 May	51	50
*P. astreoides*	Belize	1 m	2 June, 2011	3-8 June	22	6
*A. agaricites*	Belize	10 m	2 June, 2011	3-6 June	14	5
*A. tenuifolia*	Belize	1-3 m	1 June, 2011	3 June	12	2
*M. ferox*	Belize	20 m	2 June, 2011	5 June	15	1
*F. fragum*	Belize	1-3 m	9 June, 2011	11-14 June	21	6
*F. fragum*	Belize	1-3 m	10 August, 2011	12 August	20	5

^1^ Location of collection

^2^ Date colonies were collected and brought to the lab

^3^ Date first brooded larvae were released by colonies

^4^ Number of adult colonies collected for each species

^5^ Number of adult colonies that actually released brooded planulae

### Collection of Gametes, Planulae and Adults from Broadcast Spawning Species in Florida and Belize

 Gametes and planulae were collected from three and five Caribbean broadcast spawning coral species in the Florida Keys and Belize, respectively (Figure 1, Table 2). In August and September 2011, gametes were collected 2–4 hours after sunset by tenting individual colonies prior to release. Gamete bundles were returned to the boat or laboratory, allowed to break apart, and then mixed with bundles from different colonies to increase fertilization success [42]. The only exception was *Orbicella* (formerly *Montastraea* [43]) *franksi*, which was from a reef ~1.5 km south of CBC (Table 2). In this case, *O. franksi* colonies were transported to the lab and placed in individual ~18 L containers similar to the brooding species (see above). Planulae from all species were reared for between 18 hrs and 9 days and preserved in 95% ethanol prior to metamorphic competence. Gametes were also preserved separately when they were collected in excess of what was needed for crosses (Table 2, see below). Since gametes from all adults were mixed during fertilization attempts, it was impossible to track the exact parental colonies of the resulting planulae.

**Table 2:** Information for the broadcast spawning scleractinian coral species sampled in this study from Florida and Belize.

**Location**	**Species**	**Fertilization^1^**	**P/E/S^2^**	**# Par^3^**	**Parental Reef^4^**	**GPS**
**Florida**	*A. palmata*	16-Aug, 2011	P/E	1	Elbow Reef	N 25.139722°, W 80.294167°
	*A. palmata*	16-Aug, 2011	P/E	1	Horseshoe Reef	N 25.1425°, W 80.25835°
	*A. palmata*	17-Aug, 2011	P	1	Molasses Reef	N 25.01015°, W 80.37328°
	*A. palmata*	17-Aug, 2011	P	1	Horseshoe Reef	N 25.1425°, W 80.25835°
	*O. faveolata*	20-Aug, 2011	P	3-5	Horseshoe Reef	N 25.1425°, W 80.25835°
	*O. faveolata*	19-Aug, 2011	P	10-15	Looe Key	N 24.544878°, W 81.409361°
	*O. faveolata*	19-Aug, 2011	P	10-15	Alligator Reef	N 24.81285°, W 80.66945°
	*P. Strigosa*	19-Aug, 2011	P	2	Horseshoe Reef	N 25.1425°, W 80.25835°
**Belize**	*A. cervicornis*	17-Aug, 2011	P/E/S	6	CBC Reef	N 16.8025°, W 88.08194°
	*A. palmata*	16-Aug, 2011	P/E/S	3	CBC Reef	N 16.8025°, W 88.08194°
	*O. faveolata*	19-Sep, 2011	P/E/S	7	CBC Reef	N 16.8025°, W 88.08194°
	*O. franksi*	19-Aug, 2011	E/S^5^	5	CBC Wall	N 16.77972°, W 88.07528°
	*P. Strigosa*	19-Sep, 2011	P	3	CBC Reef	N 16.8025°, W 88.08194°

^1^ Date of gamete collection and fertilization

^2^ Denotes which samples were collected from each reef: Planulae (P), Eggs (E), and sperm (S).

^3^ The number of parents (# Par) utilized in the gamete cross

^4^ Parental Reef with corresponding GPS coordinates.

^5^ no viable larvae obtained from *O. franski.*

 The hypothesis that apicomplexans are transmitted horizontally in broadcast spawning coral species assumes adults in the population (including those not contributing to the gametic pool) are associated with these symbionts. Thus, it was necessary to screen adults in the population. In Florida, however, it was not possible to sample the exact colonies providing gametes for planula generation due to logistic difficulties. Instead, single polyps from the top, middle and bottom of 24 Floridian *Orbicella* (formerly *Montastraea* [43]) *faveolata* colonies were sampled at Alligator Reef (one of the sites where gametes were collected) a few days after the spawning event using the syringe technique of Correa et al. [44] and preserved in 95% ethanol. At CBC in Belize, tissue from the six *Acropora cervicornis* and three *Acropora palmata* colonies that provided gametes for crosses were preserved in CHAOS buffer [45]. Additionally, all *A. cervicornis* and *A. palmata* individuals from the same reef flat as the colonies providing gametes for crosses were sampled for other studies. From these, 30 colonies of each *Acropora* species were randomly selected to include those of reproductive age using a colony size cutoff metric of 2,500 cm^2^ and 600 cm^2^ for *A. palmata* and *A. cervicornis*, respectively. If the gamete-providing colonies were not selected as part of this random subset, they were also included, increasing the number of individuals examined to 31 and 33 for *A. palmata* and *A. cervicornis*, respectively.

### DNA extraction and Presence/Absence Screening for Apicomplexans

 Preliminary experiments determined 3–5 brooding and 20 broadcast spawning planulae consistently provided ~5–10 ng/uL of template DNA, sufficient to produce a strong amplicon via PCR with the three primer sets and thermocycling conditions (see below) employed in this study. Therefore, DNA was extracted from single batches of either 5 planulae for each adult sampled of all brooding species or 20 planulae for each conducted cross of gametes from broadcast spawning species. Additionally, the presence of apicomplexans in gametes prior to syngamy was assessed in Belize by combining and extracting DNA from ~100 eggs or all sperm collected from an individual. Most DNA extractions were done solely using 2X CTAB buffer, with tissue homogenization by pestles and bead-beating prior to phenol:chloroform extraction [46]. However, due to co-precipitation of inhibitors, all *P. astreoides* adult samples were also gel purified using Spin-X filters (Corning Costar^®^) following 2X CTAB extraction. For *Acropora* colonies from Belize, DNA was isolated using the protocol described in Levitan et al. [47]. As a control for potential contamination during DNA isolation, no-larvae controls were included during all extractions; these controls utilized all the same buffers, plastic consumables, and protocol steps except planulae or gametes were not included.

 To determine whether DNA templates were free of PCR inhibitors, the small subunit ribosomal DNA (18S rDNA) was first amplified from all samples utilizing the “universal” primers SS5 and SS3 [48], which amplifies cnidarians, *Symbiodinium*, and other eukaryotes. Reactions were conducted in 10 μL volumes containing 10 mM Tris HCL, 1.5 mM MgCls, 50 mM KCl, 0.2 mM dNTPs, 0.3 μM of each primer and 1 U of Taq polymerase [49] for 30 cycles of 94° C for 1 min, 56° C for 1 min, and 72° C for 1.5 min followed by a final 5 min extension step at 72° C. This was followed by apicomplexan screening via a presence/absence PCR based assay with the apicomplexan-specific 18S rDNA primers 18N-F2 and 18N-R1 [31,35]. Here, 10 μL reactions (as above) were conducted using a touchdown PCR protocol, starting with an initial denaturing step of 95° C for 5 min followed by 10 cycles of 94° C for 45 s, 60° C deceasing 1° C each cycle until 50° C was reached, and a extension step of 72° C for 1 min. This was immediately followed by 30 cycles of 94° C for 45 s, 50° C for 45 sec and 72° C for 1 min and a final extension at 72° C for 5 min. To ensure that these latter amplicons were derived from apicomplexan template DNA, twenty samples from the brooded planulae dataset and representative of all examined brooding species were selected by a random number generator and sequenced in the forward direction with the primer 18N-F2. As an additional test of DNA template integrity, twelve gamete or planulae samples were randomly selected from the Florida and Belize broadcast spawning species and an ~710 bp fragment of the coral mitochondrial cytochrome oxidase subunit I (COI) gene amplified utilizing the “universal” metazoan primers of Folmer et al. [50] and protocol of Craft et al. [51]. These twelve samples were sequenced using the primer LCO1490 [50]. All sequences were trimmed in Sequencher v5.0.1 prior to being submitted to GenBank’s non-redundant (nr) database using blastn [52] to identify their most similar matches. All generated sequences longer than 200 bp were submitted to GenBank under accession numbers (KF579883-KF579909). All sequences are publicly available from http://www.auburn.edu/~santosr/sequencedatasets.htm.

### Statistical Analyses

 Prevalence (i.e., calculated as the frequency at which apicomplexan DNA was detected via the PCR assay divided by the number of examined samples and expressed as a percentage [53]) was calculated, along with 95% Confidence Intervals (C.I.), using Sterne’s exact method in qp v3.0 [54]. Prevalence was compared among planulae of the brooding coral species using Fisher’s exact test as there were few samples where apicomplexans were not detected (see Results) and significance was adjusted by the Bonferroni correction . Likewise, prevalence was compared between all pairs of broadcast spawning coral species in Belize from the screenings of sperm, eggs, and/or adult colonies. Fisher’s exact tests were also utilized to determine whether apicomplexan prevalence between brooding and broadcast spawning coral species were significantly different. Specifically, comparisons were made between all brooding planulae (*n* = 89) and all sperm (*n* = 28), egg (*n* = 28) and batches of planulae (*n* = 10) from the broadcast spawning species.

## Results

### Apicomplexan Screening of Brooders from Florida and Belize

 Template DNA from 4 of 30 adult *P. astreoides* colonies collected in 2010 failed to amplify with either of two primer sets (i.e., universal [SS5/SS3] and apicomplexan-specific [18N-F2/18N-R1]). Following exclusion of these from further analyses, 96.2% of the remaining colonies (*n* = 25/26; 81.2–99.8% [95% C.I.]), including all (*n* = 14) that released planulae, tested positive for the presence of apicomplexan DNA. For *P. astreoides* planulae, apicomplexan DNA was detected via PCR in all (*n* = 14/14; 76.2–100%) batches of 5 planulae from all colonies that brooded in 2010. A similar pattern was identified in the subsequent year, with 92.0% (*n* = 46/50; 81.2–97.2%) apicomplexan prevalence in batches of planulae collected from 50 colonies and all batches (*n* = 5/5; 47.8–100%) of planulae collected from five colonies on three consecutive mornings testing positive for apicomplexan DNA. No significant differences in apicomplexan prevalence were identified between 2010 and 2011 (*P* = 0.57) or life stages (adult vs. planulae; *P* = 1.00) of *P. astreoides* prior to or following Bonferroni correction. Two and nine amplicons from the apicomplexan-specific PCR reactions were sequenced for the 2010 and 2011 larvae, respectively, (Table S1) and all 11 were most similar to that of the scleractinian coral-associated apicomplexan from Toller et al. [31]. 

 As in Florida, apicomplexan DNA was detected in all batches (*n* = 6/6; 58.9–100%) of *P. astreoides* planulae from Belize. Furthermore, batches of planulae from all colonies of four other brooding species (i.e., *A. Agaricites* [*n* = 5/5; 50.0–100%], *A. tenuifolia* [*n* = 2/2; 22.6–100%], *F. fragum* [*n* = 11/11; 73.4–100%], and *M. ferox* [*n* = 1/1; 5.0–100%]) tested positive for apicomplexans. There were no significant differences in apicomplexan prevalence between the two locations (i.e., Florida and the Belize: *P* = 0.57) or among any of the brooding species (*P* = 1.00 for all 10 pairwise comparisons). Again, sequencing of nine randomly selected amplicons generated with the apicomplexan-specific primer set were most similar to the same GenBank accession (Table S1) from Toller et al. [31]. It should be noted that no amplicons were produced from either the planulae-free extractions or negative (i.e., no template added) PCR controls with either of the two primer sets throughout this entire study.

### Apicomplexan Screening of Broadcast Spawning Species from Florida and Belize

 Similar to the brooding species, the majority of adult colonies from the broadcast spawning scleractinian coral species tested positive for apicomplexans both in the Florida Keys and Belize. For example, apicomplexans were detected in all *O. faveolata* colonies at Alligator Reef (*n* = 24/24; 86.1–100%) from at least one of the three polyps sampled from different parts of the same colony. More specifically, apicomplexan DNA was detected in 21, 22, and 22 of each of 24 samples taken across all *O. faveolata* colonies from the bottom, middle and top, respectively, and apicomplexans were detected in at least two of three sampled polyps in all but one colony (95.8%; *n* = 23/24; 78.9–99.9%). Likewise, apicomplexan DNA was detected in 87.9% (*n* = 29/33; 71.5–95.8%) of the examined *A. cervicornis* colonies from Belize, including 5 of 6 colonies contributing gametes towards the generation of planulae. The PCR assay for apicomplexans was also positive for 90.3% (*n* = 28/31; 74.5–97.3%) of *A. palmata* colonies on the same reef, including all three colonies from which gametes were collected. There was no significant difference between apicomplexan prevalence in adults among the three species (*P* ≥ 0.38 for all three pairwise comparisons).

 In contrast to adult colonies of the three examined broadcast spawning species as well as brooded planulae, apicomplexan DNA was not detected via the PCR assay from single batches of 20 planulae from the three broadcast spawning species (i.e., *A. palmata*, *O. faveolata*, and *Pseudodiploria* [formerly *Diploria* [43]] *strigosa*) of the Florida Keys. This was also true in Belize for batches of planulae from *A. cervicornis*, *A. palmata*, *P. strigosa*, and *O. faveolata*. Apicomplexan DNA was, however, detected in gametes collected from four of the Belize colonies, including sperm from a single *A. cervicornis* colony, eggs from an *A. palmata* colony, sperm from two *O. faveolata* colonies, and eggs from one of these same colonies (Table 3). Unlike with the apicomplexan-specific primer set, amplicons could be generated from the above apicomplexan “negative” samples using the “universal” 18S rDNA primers. This implies the presence of sufficient, inhibitor-free DNA template in general (e.g. coral and dinoflagellate) and an absence (or levels incapable of initiating/sustaining a PCR) of apicomplexan DNA. As an additional test of DNA template integrity, twelve samples were randomly selected from the complete sample set of broadcast spawning planulae and gametes for partial sequencing of the metazoan mitochondrial COI gene. Subsequent BLAST searches of these sequences found them to be 100% identical to GenBank accessions for scleractinian corals of their respective genera (Table S2), indicating the presence of amplifiable host DNA template.

**Table 3:** Number of instances where apicomplexan DNA was detected in gametes from broadcast spawning coral species sampled in this study from Florida or Belize.

**Location**	**Species**	**Gamete**	**Present^1^**	**Total^2^**	**95% C. I.^3^**
**Florida**	*A. palmata*	Eggs	0	2	
**Belize**	*A. palmata*	Eggs	1	3	0-86.46%
	*A. palmata*	Sperm	0	3	
	*A. cervicornis*	Eggs	0	6	
	*A. cervicornis*	Sperm	1	6	0-58.86%
	*O. faveolata*	Eggs	1	7	0-55.42%
	*O. faveolata*	Sperm	2	7	0-70.96%
	*O. franksi*	Eggs	0	10	
	*O. franksi*	Sperm	0	10	

^1^ Number of gamete samples possessing detectable apicomplexan DNA

^2^ Total number of colonies providing gametes that were tested (Total).

^3^ The 95% confidence interval (C.I.) for prevalence were calculated using Sterne’s exact method for all colonies that were PCR positive for apicomplexan DNA in at least one sample.

Statistical Comparison of Apicomplexan Prevalence in Brooding vs. Broadcast Spawning Coral Species in Belize

**Figure 2:**
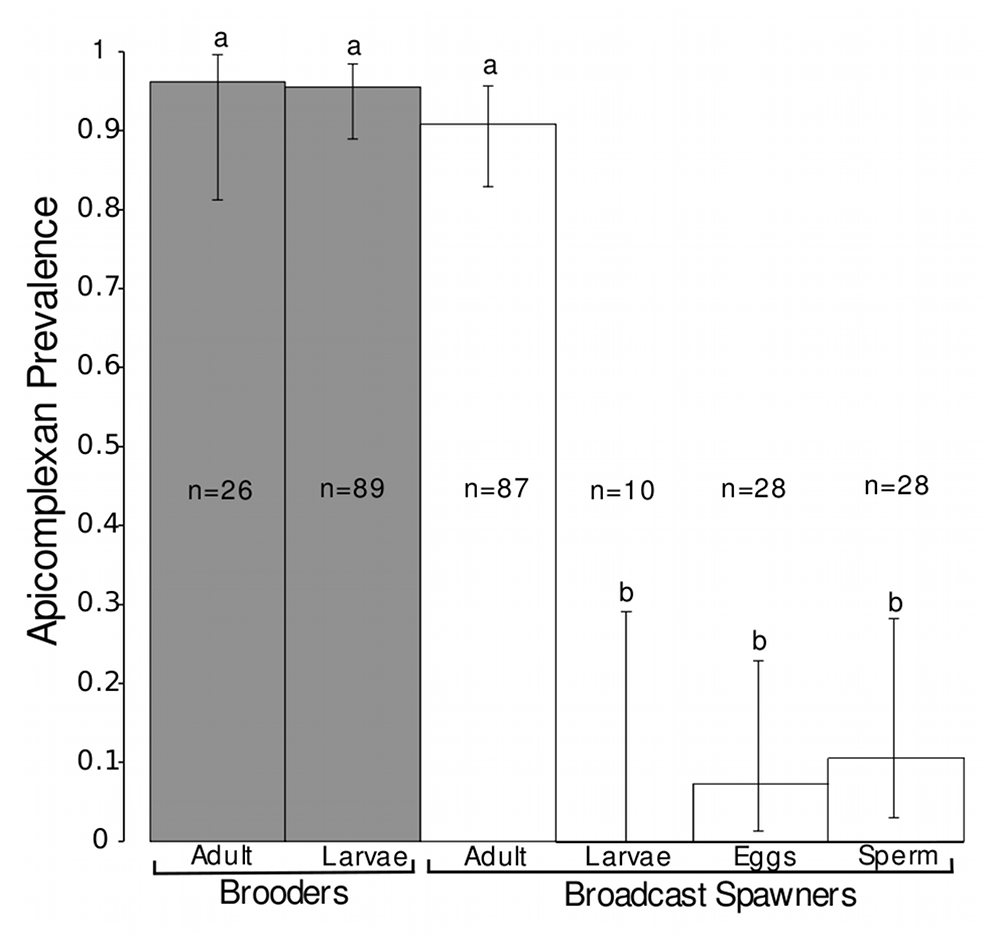
Total apicomplexan prevalence among brooding (grey bars) and broadcast spawning corals (white bars). Error bars represent 95% confidence intervals. Statistical significance via Fisher’s exact tests is noted at *P* > 0.001. Species that were included in each of the categories are as follows: Brooding adults: *Porites astreoides*. Brooding planulae: *Agaricia agaricites*, *Agaricia tenuifolia*, *Favia fragum*, *Mycetophyllia ferox*, *P. astreoides*. Broadcast spawning adults: *Acropora cervicornis*, *Acropora palmata*, *Orbicella faveolata*. Broadcast spawning planulae: *A. cervicornis*, *A. palmata*, *O. faveolata*, *Pseudodiploria strigosa*. Broadcast spawning sperm: *A. cervicornis*, *A. palmata*, *O. faveolata*, *Orbicella franksi*. Broadcast spawning eggs: *A. cervicornis*, *A. palmata*, *O. faveolata*, *O. franksi*.

 While apicomplexan prevalence was not significantly different between adult colonies and planulae of the brooding scleractinian coral species (*P* = 1.00; see above), there was a significant difference between these life stages in the broadcast spawning scleractinian coral species (*P* = 3.5 x 10^-9^; Figure 2). Overall, a significant difference in apicomplexan prevalence was also apparent between planulae of the Caribbean brooding and broadcast spawning species examined here (*P* = 6.4x10^-11^; Figure 2).

 No significant difference was identified between all broadcast spawning species when comparing among gamete samples (Table 4). Given this, cases of apicomplexan-positive results per category were summed to compare total prevalence between planulae and/or gametes from all brooding and broadcast spawning scleractinian coral species (i.e., brooded planulae [total prevalence = 95.5%; *n* = 85/89, 88.9-98.5%], broadcasted eggs [7.1%; *n* = 2/28, 1.3-22.9%], broadcasted sperm [10.7%; *n* = 3/28, 3.0-28.2%], and broadcasted planulae [0%, *n* = 0/10, 0.0-29.1%]). Significant differences in the prevalence of apicomplexan DNA were identified between brooded planulae relative to eggs (*P* < 2.2 x 10^-16^), sperm (*P* < 2.2 x 10^-16^), and planulae (*P* = 6.4 x 10^-11^) of the broadcast spawning scleractinian coral species examined here (Figure 2). 

**Table 4:** Pairwise comparisons of apicomplexan prevalence between eggs (top) and sperm (bottom) of broadcast spawning scleractinian coral species sampled in this study from Belize.

	*O. faveolata*	*O. franksi*	*A. cervicornis*	*A. palmata*
*O. faveolata*	XXX	1.000^1^	1.000	1.000
*O. franksi*	0.205	XXX	1.000	0.177
*A. cervicornis*	1.000	0.483	XXX	0.258
*A. palmata*	1.000	1.000	1.000	XXX

^1^ *P*-values from Fisher’s exact test are presented. None were significant before or after Bonferroni correction.

## Discussion

 Here, apicomplexan DNA was detected from nearly all adult Caribbean scleractinian colonies spanning multiple species and years as well as two geographic locations. Prevalence among colonies was high (88–100%) and consistent with histological [29,30] and molecular [31,35] studies, implying apicomplexans are common symbionts of Caribbean scleractinian corals. Several mechanisms could explain the ubiquitous distribution of these symbionts among diverse coral species. For example, vertical transmission in brooding coral species could contribute to high prevalence as apicomplexans were detected in the majority (96%) of planulae sampled from five species. On the other hand, apicomplexan DNA was not detected in planulae, and only at low (7–11%) frequencies in gamete samples, from five broadcast spawning coral species. This suggests apicomplexans associated with broadcast spawning scleractinian species are likely acquired horizontally, such as from the sediment, the water-column, paratenic (i.e. transport) and/or intermediate hosts, or via other avenues.

 The PCR assay employed here is an indirect method of assessing apicomplexans within coral larvae and false positives and negatives are possible [55]. For example, false positives might arise from contamination or non-specific priming during PCR. Notably, all DNA extraction and PCR controls were negative in this study. This implies contamination was not likely a contributing factor to the high incidence of apicomplexan recovery from the planulae of the brooding coral species as well as adults of *P. astreoides* or the broadcast spawning coral species. As for primer specificity to apicomplexan 18S rDNA, sequences generated from 20 randomly selected amplicons here, as well as 100 randomly selected amplicons from a previous study [35], were most similar to a coral-associated apicomplexan they were designed to target [31]. False negatives are also a possibility, as a minimal DNA template concentration is needed in to order to initiate a PCR. As such, we cannot discount whether apicomplexans were present in broadcast spawning planulae at abundances below such a threshold. However, total DNA template concentrations were standardized between brooded and spawned planulae and both were subjected to identical PCR protocols utilizing the same primer sets. Lastly, while mismatches between the primers and DNA template present in the larvae of the broadcast spawners would produce a similar pattern of apicomplexan “negative”, this seems highly unlikely, particularly since adults of the same species are near ubiquitously apicomplexan “positive”. 

### Vertical Transmission of Apicomplexans in Brooding Corals

 Apicomplexan symbionts were detected among brooded planulae from a majority (*n* = 66/70, 94.3%) of *P. astreoides* colonies sampled in two different years on Floridian and Belizean reefs. Furthermore, a nearly identical pattern was identified in four additional brooding scleractinian coral species in Belize. This represents, to the best of our knowledge, the first evidence in support of vertical transmission of apicomplexans among brooding scleractinian coral species along with extending the host range of these symbionts to *F. fragum*, *A. tenuifolia*, and *M. ferox*. Notably, the presence of apicomplexans in this early life stage appears temporally consistent over a reproduction event since these symbionts were detected from the brooded planulae of *P. astreoides* up to three days following initial release.

 Why might apicomplexans be vertically transmitted in brooding scleractinian coral species? Given that planulae of brooding corals are physically large [56], this may simply be a function of sufficient volumetric space for the storage and passage of symbionts and it is not uncommon to find such planulae provisioned with a variety of other symbionts [20]. For example, *Symbiodinium* was previously reported within the planulae of the five brooding species examined in this study [41,57–59] and bacterial symbionts [23] and ARLs [33] have been similarly documented within larvae of *P. astreoides*. Thus, vertical transmission of apicomplexans for these brooding scleractinian coral species is parsimonious with both the inheritance pattern of other symbionts as well as with other host-apicomplexan systems in general [60,61]. In these latter cases, apicomplexans are directly passed to zygotes and larvae of other aquatic host species [62,63] and in one case, the eugregarine *Diplauxis hatti* is capable of arresting development to couple its reproduction with that of its annelid host [62]. It remains to be determined, however, whether these or other brooding scleractinian coral species have the ability to acquire apicomplexan symbionts via horizontal transmission as well.

### Horizontal Transmission of Apicomplexans in Broadcast Spawners

 In contrast to the five brooding scleractinian coral species examined here, apicomplexans were not detected from the planulae of the four broadcast spawning scleractinian coral species surveyed in Florida or Belize. Although viable planulae were not obtained from *O. franski*, apicomplexans were not detected in gametes from this species, consistent with this pattern. While there is a possibility that broadcast spawning planulae harbor apicomplexans below the threshold detection limit of the employed PCR assay, such a situation is unlikely as initial screenings of DNA extractions pooling up to 100 broadcast spawning planulae were also apicomplexan “negative” (data not shown). Given that most (*n* = 106/114, 92.9%) adult colonies in this and other studies [30,31,35] harbor these symbionts, broadcast spawning scleractinian coral species most likely acquire apicomplexans via horizontal transmission at a post-planula life stage. Again, this mirrors the transmission mode of other symbionts from broadcast spawning corals [20,24], including *Symbiodinium* and bacterial symbionts for the five host species in this study [24,64–67]. In the latter case, Eubacteria were not found within the tissues of corals until after settlement occurred [24]. 

 Horizontal transmission involves the encounter of a suitable host and symbiont, either via environmental mechanisms and/or through paratenic/intermediate hosts. For example, apicomplexans were detected in a few (*n* = 5/54, 9.3%) gametic samples from three of the broadcast spawning species and this may represent one mechanism of horizontal transmission. Specifically, all five broadcast spawning species in this study are hermaphroditic, releasing positively buoyant gamete bundles that break apart and cross-fertilize in surface waters [68]. These bundles are bound in a mucous coating [69] that may trap apicomplexan cells upon release from the parental colony. Consequently, transmission of apicomplexans between host individuals might be facilitated by corallivorous fishes as a consequence of consuming broadcasted gamete bundles [21,70] and defecation of viable cells, similar to the fish fecal transmission hypothesized for *Symbiodinium* [71–73]. Furthermore, oocysts (i.e., resting stages) could be transmitted directly among colonies trapped within mucous layers of corallivorous fish mouths, as previously hypothesized by Upton and Peters [30]. 

 Regardless of transmission route, source populations of apicomplexans are required to infect apparently aposymbiotic planulae, be it from benthic substrates, the water column [74], and/or established colonies. For the former case, *Symbiodinium* capable of forming symbiotic relationships [75] as well as the alveolate parasites of bivalves, *Perkinsus* spp. [76–78], have been recovered from benthic reservoirs. Thus, apicomplexans, either as physiologically active cells or oocysts, may reside in the benthos until being horizontally acquired by newly-settled planulae of broadcast spawning coral species. Notably, the apicomplexan-related lineage (ARL) V has only been found in coral tissue and not adjacent water, macroalgal, or benthic samples [34], suggesting the persistence of coral-associated apicomplexans in the environment may be short-lived. Adult colonies also provide a potentially large source pool of apicomplexans to newly-settled and aposymbiotic planulae. In this context, corals grow clonally and some colonies can persist for hundreds of years [79], with consistent apicomplexan prevalence across time [35]. Furthermore, colony fragmentation, which can propagate clones of scleractinian corals like *A. palmata* and *A. cervicornis* on a reef [80,81], also increases the number of individuals harboring these symbionts. High estimates of clonality at CBC for the two *Acropora* species (Methods S1, Table S3) are consistent with those from reefs in the Caribbean Sea [82,83] and nearly all adult *Acropora* spp. examined here harbored apicomplexans. Clonal transmission of symbionts has been documented in other systems where the host undergoes asexual reproduction, such as the bacterial symbionts of aphids and flatworms [84,85]. Additionally, myxozoan parasites are propagated in freshwater bryozoans by fragmentation of the host [86], leading to high prevalence [87]. Thus, clonal propagation by a scleractinian host could contribute towards the high apicomplexan prevalence seen among adult colonies while simultaneously increasing the source pool of these symbionts for future generations.

## Conclusions and Future Directions

 This study demonstrates that Caribbean scleractinian corals likely acquire their apicomplexan symbionts via different routes of transmission (i.e., vertically and horizontally) depending on the reproductive mode of the host species. While this information furthers our knowledge regarding these apparently widespread, but under-recognized, symbioses spanning the Caribbean Sea [29,30,32] as well as the Eastern Pacific Ocean [31], numerous questions remain to be addressed, including: Are intermediate/paratenic hosts involved in these relationships and, if so, do they transport these symbionts between and among reefs? When apicomplexans are acquired by broadcast spawning scleractinian coral species, does this occur following planula settlement and metamorphosis or as adult colonies? Do coral-associated apicomplexans exhibit host specificity or are they “generalists” of both brooding and broadcast spawning species? Finally, the exact nature of the interaction between these apicomplexans and their coral hosts remains to be determined. For example, plastid DNA from the apicomplexan related lineage ARL-V has been detected in numerous scleractinian corals, including *P. astreoides* planulae [33,34]. Interestingly, these symbionts were not found at depth (i.e., 20 m), prompting the yet untested hypothesis that they may have photosynthetic capabilities. Unfortunately, since different molecular markers were utilized, it is unclear whether the apicomplexans detected here and ARL-V are (or belong to) the same group of organisms, as was recently hypothesized [36]. Thus, further characterization of coral-associated apicomplexans and ARLs will be required to elucidate the relationship between these enigmatic taxa.

## Supporting Information

Methods S1 **Apicomplexan Prevalence and Clonality of *Acropora* sp. in Belize.**
(DOC)

Table S1 **BLAST results for planulae of brooding species sampled from Florida (Fla) and Belize (Bel) amplified with the 18S rDNA apicomplexan-specific primers.** The host species (Query), the query length (in base pairs), description and accession number of the best BLAST hit (Top-Hit) are provided. Percent identity (% Id) between the query and hit and E-values are also provided.
(DOC)

Table S2 **BLAST results for planulae (**P**), Eggs (**E**) or sperm (**S**) of broadcast spawning coral species sampled from Florida (Fla) and Belize (Bel) and amplified with the 18S rDNA apicomplexan-specific primers.** The host species (Query), the query length (in base pairs), description and accession number of the best BLAST hit (Top-Hit) are provided. Percent identity (% Id) between the query and hit and E-values are also provided.
(DOC)

Table S3 **Apicomplexan prevalence among clones of *A. palmata* and *A. cervicornis*.** For each clone, the number of ramets (i.e. individuals per clone; N ramet) and ramets testing positive for apicomplexans (N pos) is given along with the 95% confidence interval (C.I.).
(DOC)
