# Supplementary material for: Tracking Transmission of Apicomplexan Symbionts in Diverse Caribbean Corals
Source: PLoS One. 2013 Nov 19;8(11):e80618. doi: 10.1371/journal.pone.0080618 (PMC3833926; doi:10.1371/journal.pone.0080618)
Supplement: Methods S1 — Apicomplexan Prevalence and Clonality of Acropora sp. in Belize. (DOC) [file pone.0080618.s001.doc]

**Supplementary Methods and Results**

Assessing Clonality of Parental *Acropora* spp. in Belize

Both *A. cervicornis* and *A. palmata* commonly undergo clonal propagation by fragmentation . To assess the clonality among the adult colonies examined here, individuals were genotyped at three polymorphic microsatellite loci: 181, 182, and 187 . All samples were amplified using the protocol of Fogarty et al. and alleles separated on an ABI 3100 xl (Applied Biosystems®) capillary sequencer. Allelic sizes were determined using Genescan v3.1 and Genotyper v3.7 software (Applied Biosystems). The probability of identity (P.I.), which represents the potential that identical and shared genotypes are by chance and not common descent , was calculated for each of the three loci in Gimlet v1.3.3 . The combined probability of identifying such clonemates utilizing three loci was calculated by multiplying the three individual locus probabilities . The number of genets (N_g_) and ratio of genets to ramets (N; i.e., the sample size examined) also were calculated.

Apicomplexan Prevalence and Clonality of *Acropora* sp. in Belize

Utilizing three microsatellite loci, the biased and unbiased P.I. between colonies of *A. cervicornis* were 1.36x10^-2^ and 3.65x10^-2^, respectively, while the same estimates for *A. palmata* were 3.37x10^-4^ and 1.37x10^-4^, respectively. Thus, these microsatellite loci can discriminate between ramets of both species. From the 33 *A. cervicornis* and 31 *A. palmata* individuals (N) sampled, five (genet to ramet [N_g_/N] ratio= 0.156) and 17 (N_g_/N = 0.548) unique genets (N_g_) were identified for *A. cervicornis* and *A. palmata*, respectively. For *A. cervicornis*, 87.9% (*n* = 29/33) of the examined individuals were ramets belonging to a single genet (Supplementary Table 3). In total, all (*n* = 5/5) *A. cervicornis* and 82.3% of the *A. palmata* (*n* = 14/17) genets tested positive for apicomplexan DNA. Interestingly, all ramets of *A. palmata* testing negative for apicomplexans (*n* = 3/17) represented genets comprised of a single ramet (*n* = 1). However, there was no statistical difference between the apicomplexan prevalence of genets represented once and those comprised of multiple ramets (*P* = 0.53).

1. Lirman D (2000) Fragmentation in the branching coral *Acropora palmata* (Lamarck): growth, survivorship, and reproduction of colonies and fragments. J Exp Mar Biol Ecol 251: 41-57.

2. Tunnicliffe V (1981) Breakage and propagation of the stony coral *Acropora cervicornis*. Proc Natl Acad Sci U S A 78: 2427-2431.

3. Baums IB, Hughes CR, Hellberg ME (2005) Mendelian microsatellite loci for the Caribbean coral *Acropora palmata*. Mar Ecol Prog Ser 288: 115-127.

4. Fogarty ND, Vollmer SV, Levitan DR (2012) Weak prezygotic isolating mechanisms in threatened Caribbean *Acropora* corals. PLoS One 7: e30486.

5. Lasker HR, Coffroth MA (1999) Responses of clonal reef taxa to environmental change. Amer Zool 39: 92-103.

6. Valiére N (2002) GIMLET: a computer program for analysing genetic individual identification data. Mol Ecol Notes 2: 377-379.

7. Baums IB, Miller MW, Hellberg ME (2005) Regionally isolated populations of an imperiled Caribbean coral, *Acropora palmata*. Mol Ecol 14: 1377-1390.
