# Supplementary material for: Tracking Transmission of Apicomplexan Symbionts in Diverse Caribbean Corals
Source: PLoS One. 2013 Nov 19;8(11):e80618. doi: 10.1371/journal.pone.0080618 (PMC3833926; doi:10.1371/journal.pone.0080618)
Supplement: Table S1 — BLAST results for planulae of brooding species sampled from Florida (Fla) and Belize (Bel) amplified with the 18S rDNA apicomplexan-specific primers. The host species (Query), the query length (in base pairs), description and accession number of the best BLAST hit (Top-Hit) are provided. Percent identity (% Id) between the query and hit and E-values are also provided. (DOC) [file pone.0080618.s002.doc]

| **Fla/Bel** | **Query** | **Length (bp)** | **E-Values** | **% Id** | **Top-Hit** | **Description** |
| --- | --- | --- | --- | --- | --- | --- |
| Bel | *A. agaricites* | 589 | 0 | 100% | AF238264 | Coral symbiont (18S rDNA) |
| Bel | *A. tenuifolia* | 728 | 0 | 99% | AF238264 | Coral symbiont (18S rDNA) |
| Bel | *F. fragum* | 681 | 0 | 100% | AF238264 | Coral symbiont (18S rDNA) |
| Bel | *F. fragum* | 625 | 0 | 100% | AF238264 | Coral symbiont (18S rDNA) |
| Bel | *F. fragum* | 734 | 0 | 100% | AF238264 | Coral symbiont (18S rDNA) |
| Bel | *F. fragum* | 234 | 2.0x10^-104^ | 97% | AF238264 | Coral symbiont (18S rDNA) |
| Fla | *P. astreoides* | 716 | 0 | 99% | AF238264 | Coral symbiont (18S rDNA) |
| Fla | *P. astreoides* | 122 | 7.0x10^-54^ | 99% | AF238264 | Coral symbiont (18S rDNA) |
| Fla | *P. astreoides* | 576 | 0 | 99% | AF238264 | Coral symbiont (18S rDNA) |
| Fla | *P. astreoides* | 220 | 5.0x10^-105^ | 99% | AF238264 | Coral symbiont (18S rDNA) |
| Fla | *P. astreoides* | 648 | 0 | 98% | AF238264 | Coral symbiont (18S rDNA) |
| Fla | *P. astreoides* | 127 | 1.0x10^-56^ | 99% | AF238264 | Coral symbiont (18S rDNA) |
| Fla | *P. astreoides* | 637 | 0 | 97% | AF238264 | Coral symbiont (18S rDNA) |
| Fla | *P. astreoides* | 638 | 0 | 97% | AF238264 | Coral symbiont (18S rDNA) |
| Fla | *P. astreoides* | 735 | 0 | 99% | AF238264 | Coral symbiont (18S rDNA) |
| Fla | *P. astreoides* | 126 | 4.0x10^-56^ | 99% | AF238264 | Coral symbiont (18S rDNA) |
| Fla | *P. astreoides* | 128 | 3.0x10^-57^ | 99% | AF238264 | Coral symbiont (18S rDNA) |
| Bel | *M. ferox* | 750 | 0 | 99% | AF238264 | Coral symbiont (18S rDNA) |
| Bel | *P. astreoides* | 548 | 0 | 99% | AF238264 | Coral symbiont (18S rDNA) |
| Bel | *P. astreoides* | 142 | 6.0x10^-65^ | 99% | AF238264 | Coral symbiont (18S rDNA) |

|  |  |  |  |  |  |  |
| --- | --- | --- | --- | --- | --- | --- |
