# Supplementary material for: Tracking Transmission of Apicomplexan Symbionts in Diverse Caribbean Corals
Source: PLoS One. 2013 Nov 19;8(11):e80618. doi: 10.1371/journal.pone.0080618 (PMC3833926; doi:10.1371/journal.pone.0080618)
Supplement: Table S2 — BLAST results for planulae (P), Eggs (E) or sperm (S) of broadcast spawning coral species sampled from Florida (Fla) and Belize (Bel) and amplified with the 18S rDNA apicomplexan-specific primers. The host species (Query), the query length (in base pairs), description and accession number of the best BLAST hit (Top-Hit) are provided. Percent identity (% Id) between the query and hit and E-values are also provided. (DOC) [file pone.0080618.s003.doc]

| **Fla/Bel** | **P/E/S** | **Query** | **Length (bp)** | **E-Values** | **% Id** | **Top-Hit** | **Description** |
| --- | --- | --- | --- | --- | --- | --- | --- |
| Bel | S | *A. cervicornis* | 283 | 1.0x10^-143^ | 100% | AY451340 | *Acropora cervicornis* (COI) |
| Bel | E | *A. cervicornis* | 556 | 0 | 100% | AY451340 | *Acropora cervicornis* (COI) |
| Bel | E | *A. cervicornis* | 552 | 0 | 100% | AY451340 | *Acropora cervicornis* (COI) |
| Bel | E | *A. palmata* | 616 | 0 | 100% | AY451341 | *Acropora palmata* (COI) |
| Bel | P | *P. strigosa* | 581 | 0 | 100% | AY451349 | *Diploria strigosa* (COI) |
| Fla | P | *O. faveolata* | 377 | 0 | 100% | HQ203282 | *Montastraea annularis* (COI) |
| Bel | E | *O. faveolata* | 522 | 0 | 100% | HQ203283 | *Montastraea annularis* (COI) |
| Bel | P | *O. faveolata* | 549 | 0 | 100% | HQ203284 | *Montastraea annularis* (COI) |
| Bel | E | *O. faveolata* | 454 | 0 | 100% | HQ203285 | *Montastraea annularis* (COI) |
| Fla | P | *O. faveolata* | 509 | 0 | 100% | HQ203286 | *Montastraea annularis* (COI) |
| Fla | P | *O. faveolata* | 554 | 0 | 100% | HQ203287 | *Montastraea annularis* (COI) |
| Bel | S | *O. franksi* | 429 | 0 | 100% | HQ203288 | *Montastraea annularis* (COI) |
