# Supplementary material for: Tracking Transmission of Apicomplexan Symbionts in Diverse Caribbean Corals
Source: PLoS One. 2013 Nov 19;8(11):e80618. doi: 10.1371/journal.pone.0080618 (PMC3833926; doi:10.1371/journal.pone.0080618)
Supplement: Table S3 — Apicomplexan prevalence among clones of A. palmata and A. cervicornis. For each clone, the number of ramets (i.e. individuals per clone; N ramet) and ramets testing positive for apicomplexans (N pos) is given along with the 95% confidence interval (C.I.). (DOC) [file pone.0080618.s004.doc]

| ***Acropora palmata* (n=31)** | | | |  |  | |  |  |
| --- | --- | --- | --- | --- | --- | --- | --- | --- |
| **Clone** | **N ramet** | **N pos** | **Prevalence** | **95% C.I.** |  | | | |
| 1 | 8 | 8 | 1 | 0.64-1 |  | | | |
| 2 | 4 | 4 | 1 | 0.47-1 |  | | | |
| 3 | 2 | 2 | 1 | 0.22-1 |  | | | |
| 4 | 2 | 2 | 1 | 0.22-1 |  | | | |
| 5 | 2 | 2 | 1 | 0.22-1 |  | | | |
| 6 | 1 | 1 | 1 | 0.05-1 |  | | | |
| 7 | 1 | 1 | 1 | 0.05-1 |  | | | |
| 8 | 1 | 1 | 1 | 0.05-1 |  | | | |
| 9 | 1 | 1 | 1 | 0.05-1 |  | | | |
| 10 | 1 | 1 | 1 | 0.05-1 |  | | | |
| 11 | 1 | 1 | 1 | 0.05-1 |  | | | |
| 12 | 1 | 0 | 0 | 0-0.95 |  | | | |
| 13 | 1 | 1 | 1 | 0.05-1 |  | | | |
| 14 | 1 | 0 | 0 | 0-0.95 |  | | | |
| 15 | 1 | 0 | 0 | 0-0.95 |  | | | |
| 16 | 1 | 1 | 1 | 0.05-1 |  | | | |
| 17 | 1 | 1 | 1 | 0.05-1 |  | | | |
| ***Acropora cervicornis* (n=33)** | | | |  |  | | | |
| **Clone** | **N ramet** | **N pos** | **Prevalence** | **95% C.I.** | |  | | |
| 1 | 29 | 25 | 0.862 | 0.69-0.95 | |  | | |
| 2 | 1 | 1 | 1 | 0.05-1 | |  | | |
| 3 | 1 | 1 | 1 | 0.05-1 | |  | | |
| 4 | 1 | 1 | 1 | 0.05-1 | |  | | |
| 5 | 1 | 1 | 1 | 0.05-1 | |  | | |
